# A Perspective on the Prowaste Concept: Efficient Utilization of Plastic Waste through Product Design and Process Innovation

**DOI:** 10.3390/ma7075385

**Published:** 2014-07-23

**Authors:** Antonio Greco, Mariaenrica Frigione, Alfonso Maffezzoli, Alessandro Marseglia, Alessandra Passaro

**Affiliations:** 1 Department of Innovation Engineering, University of Salento, Via per Arnesano, 73100 Lecce, Italy; E-Mails: mariaenrica.frigione@unisalento.it (M.F.); alfonso.maffezzoli@unisalento.it (A.M.); 2 CETMA Consortium, S.S.7 Km.706+030, 72100 Brindisi, Italy; E-Mails: alessandro.marseglia@cetma.it (A.M.); alessandra.passaro@cetma.it (A.P.)

**Keywords:** advanced application of recycled mixed plastics, reinforcement, adhesion, mechanical properties

## Abstract

This work is aimed to present an innovative technology for the reinforcement of beams for urban furniture, produced by in-mold extrusion of plastics from solid urban waste. This material, which is usually referred to as “recycled plastic lumber”, is characterized by very poor mechanical properties, which results in high deflections under flexural loads, particularly under creep conditions. The Prowaste project, founded by the EACI (European Agency for Competitiveness and Innovation) in the framework of the Eco-Innovation measure, was finalized to develop an innovative technology for selective reinforcement of recycled plastic lumber. Selective reinforcement was carried out by the addition of pultruded glass rods in specific positions with respect to the cross section of the beam, which allowed optimizing the reinforcing efficiency. The reinforcement of the plastic lumber beams with pultruded rods was tested at industrial scale plant, at Solteco SL (Alfaro, Spain). The beams obtained, characterized by low cost and weight, were commercialized by the Spanish company. The present paper presents the most relevant results of the Prowaste project. Initially, an evaluation of the different materials candidates for the reinforcement of recycled plastic lumber is presented. Plastic lumber beams produced in the industrial plant were characterized in terms of flexural properties. The results obtained are interpreted by means of beam theory, which allows for extrapolation of the characteristic features of beams produced by different reinforcing elements. Finally, a theoretical comparison with other approaches which can be used for the reinforcement of plastic lumber is presented, highlighting that, among others, the Prowaste concept maximizes the stiffening efficiency, allowing to significantly reduce the weight of the components.

## 1. Introduction

Although in recent years great attention has been given to the production of objects using recycled plastic materials [1,2,3,4,5], the poor quality of objects made from mixed recycled plastics, and the costs associated with processes capable of reducing impurities, place considerable constraints on the economic viability of recycling of plastics in general [6,7]. Most low density polyethylene (LDPE) coming from solid urban waste is processed by means of a process called the “in-mold extrusion”, or “intrusion” process. The products obtained by this technology are used to replace wood in outdoor applications, owing to their better resistance to environmental degradation. These materials are usually referred to as “recycled plastic lumber” (RPL), and are widely used in marine and high humidity environments [8]. The poor compatibility of the different polymers present in plastic waste, together with the contamination by non-polymeric materials (above all paper), results in products with poor mechanical properties.

Usually, RPL is used for the production of high aspect ratio beams, subjected to one-directional bending forces. Under such conditions, a very efficient reinforcement of the beams can be attained by the introduction of rigid rods near the upper and lower surfaces of the beam, which can be readily achieved in continuous extrusion processes through introduction of the appropriate features in the die, and continuously feeding of the reinforcing rods [9,10,11]. Recently, it was demonstrated that a similar approach can be readily adapted to the in mold extrusion process [12], as well as to other closed mold processes, such as rotational molding [13]. The resulting product is characterized by a strong anisotropy, since the reinforcing elements are placed in the zones of the beam subjected to the higher stresses. The process has therefore been adapted for the production of reinforced RPL beams on an industrial plant, thanks to the grant Eco-Innovation promoted by EACI (European Agency for Competitiveness and Innovation). The project Prowaste (Efficient utilization of Plastic Waste through Product Design and Process Innovation) was promoted by a team of partners deeply involved in the recycling and reuse chain.

In the present work, the selective reinforcement strategy used for enhancing the mechanical properties of RPL beams, and its adaptation for the production of components on an industrial plant, is presented. A preliminary selection of different materials to be used as reinforcing elements was made, based on the evaluation of the mechanical properties, costs, weight and potential durability. Reinforced beams were then produced in an industrial plant, and characterized with respect to stiffness and creep behavior of the beam, as well as the pullout resistance of the rods from the beam. Finally, the potential advantages of the Prowaste concept compared to other more conventional processes is presented.

### 1.1. Prowaste Concept

**Figure 1:**
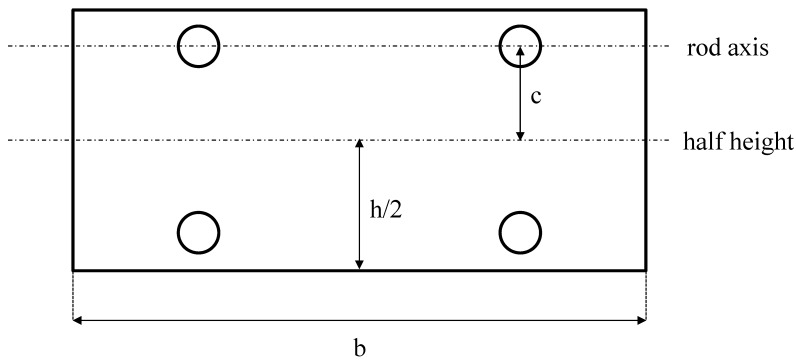
Layout of reinforcing rods.

The Prowaste concept is based on the stiffness enhancement of RPL beams by the addition of continuous rods, selectively placed at specific positions on the cross section of the beam. A possible layout of reinforcing rods for a rectangular cross section of the beam is reported in Figure 1. Reinforcement is achieved by embedding rods, characterized by a cumulative cross sectional area equal to A_ROD_, at a distance c from the half height of the cross section of the beam. Rods are disposed symmetrically with respect to the half-height of the beam, in order to avoid thermal distortions. The flexural stiffness of the reinforced RPL beam is defined as the ratio between the applied force F and the maximum deflection at half length of the beam, *v*_max_. In the case of a simply supported beam, the stiffness K is given by:

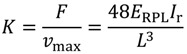
(1)
where *E*_RPL_ is the elastic modulus of the RPL, and *I*_R_ is the moment of inertia of the reinforced cross section with respect to its neutral axis. The moment of inertia depends on the beam geometry, the properties of the constituent materials, and the rods layout. For the layout reported in Figure 1, it can be calculated as:

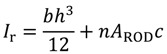
(2)
where *n* is the ratio between the modulus of the reinforcing rods, *E*_ROD_, and *E*_RPL_. The reinforcing efficiency of the rods increases as the distance between the rod position and the half height of the beam is increased. The moment of inertia of unreinforced RPL is simply obtained by Equation (2) neglecting the last term on the right hand side.

Efficient stiffening can be attained only is a full adhesion between the RPL and reinforcement is preserved. The capability of load transfer between the RPL and the rod can be estimated by considering the shear stress at the interface:

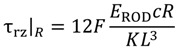
(3)
where *R* is the radius of the rods. When the shear stress at the interface overcomes the adhesion strength, debonding at the interface causes loss of the stiffening effect.

Different materials can be used to reinforce RPL beams. Among these, the materials reported in Table 1 have been evaluated. As noted in Table 1, the cost of pultruded carbon reinforced rods or aluminum rod is much higher than that of other materials, and is not compatible with the expected low cost of the products obtained from recycled plastic. The glass reinforced rods give a lower contribution to stiffness, but have a lower weight and cost compared to stainless steel. For such a reason, the pultruded glass reinforced rods were chosen to produce reinforced RPL beams. Two different types of matrices were evaluated: thermoplastic and thermoset matrices. The pultruded rods based on the thermoset matrix are characterized by an higher glass fiber content, and therefore by an higher modulus, and a lower cost compared to thermoplastic matrix based rods. Nevertheless, the higher compatibility between the thermoplastic matrix and RPL is expected to positively affect the adhesion properties, as will be better discussed in the next section.

**Table 1:** Physical properties and estimated costs of reinforcing rods.

Type of reinforcing rod	Cost (€/m) for a 3 mm Diameter Rod	Cost (€/dm^3^)	Flexural Modulus (GPa)	Density (Kg/dm^3^)
Inox steel rods	0.35	49.5	210	7.8
Aluminum rods	1.5	212	70	2.7
Glass reinforced pultruded rods based on thermosetting matrix	0.06	8.5	50	2.2
Glass reinforced pultruded rods based on thermoplastic matrix	0.3	42.5	15	1.6
Carbon reinforced pultruded rods	3	424	70	1.3

### 1.2. Production of Selectively Reinforced RPL Beams on an Industrial Plant

**Figure 2:**
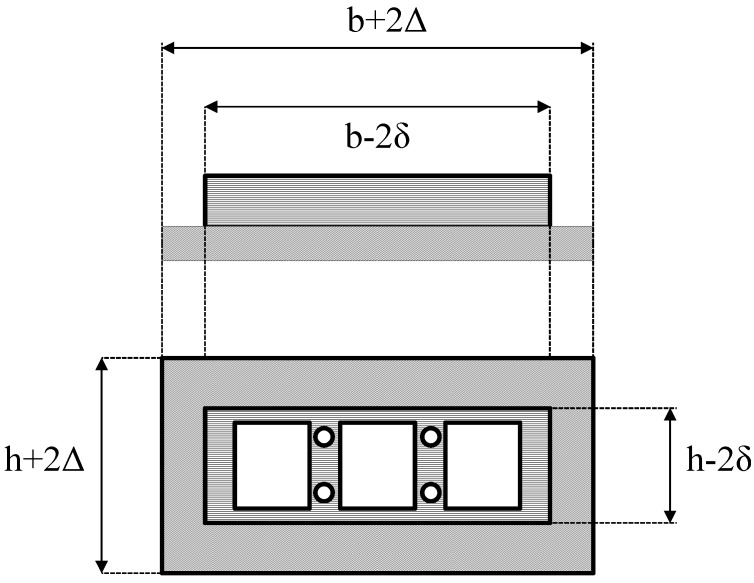
Schematic drawing of the front mask.

**Figure 3:**
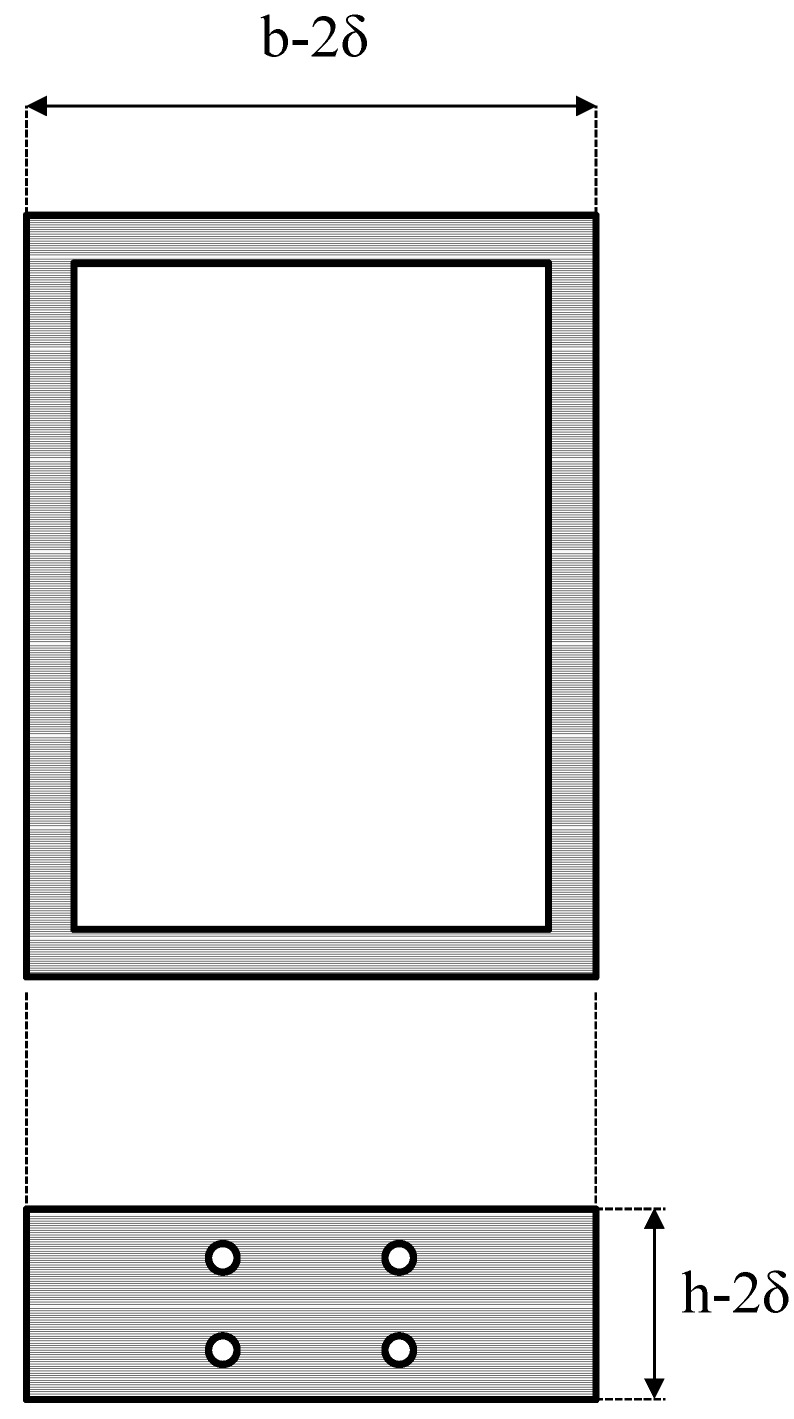
Schematic drawing of the sliding guide.

In order to incorporate the rods in the polymer mass during the in mold extrusion process, two metallic parts were properly designed and built. In the framework of the Prowaste project, Masmec SPA (Modugno, Italy) was in charge of the design of the two metallic parts. The first part is a “mask”, which is placed on the front of the mold (the surface closer to the extruder). A schematic drawing of this part is reported in Figure 2. The internal frame, depicted by horizontal hatching in Figure 2, is such that it fits inside the mold (its dimensions are (*h* − 2δ) × (*b* − 2δ), being δ a tolerance of about 0.5 mm). This frame has four small circular holes, which are required to position the rods, and three large square openings, which act as channels for the feeding of the polymer melt into the mold. The frame is provided with a screwing system, which allows blocking the rods in correspondence of the four small holes. The external frame, depicted by oblique hatching in Figure 2, is a flange which stops the mask from sliding forward during mold filling (its dimension are (*h* + 2Δ) × (*b* + 2Δ), being Δ about 10 mm). The second part is a “guide”, reported in Figure 3, which is placed inside the mold in proximity of the “mask” at the beginning of mold filling cycle and capable of sliding inside the mold as the molten polymer fills it. The outer dimension of the guide is such that is fits inside the mold (its dimensions are equal to those of the internal frame of the mask). The four holes are necessary to place the rods. Both components were made with 5 mm thick stainless steel.

**Figure 4:**
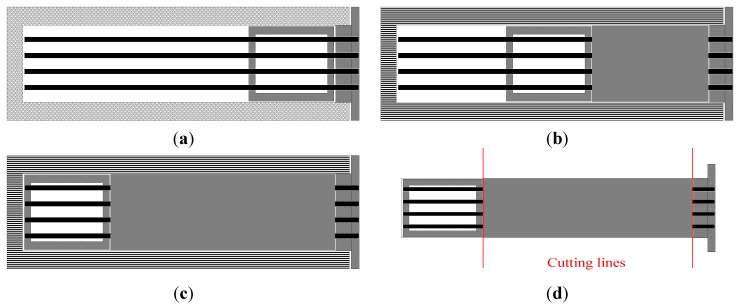
Scheme of the in mold extrusion process with selective reinforcement. (**a**) empty mold; (**b**) mold filling; (**c**) end of filling; (**d**) part extraction.

**Figure 5:**
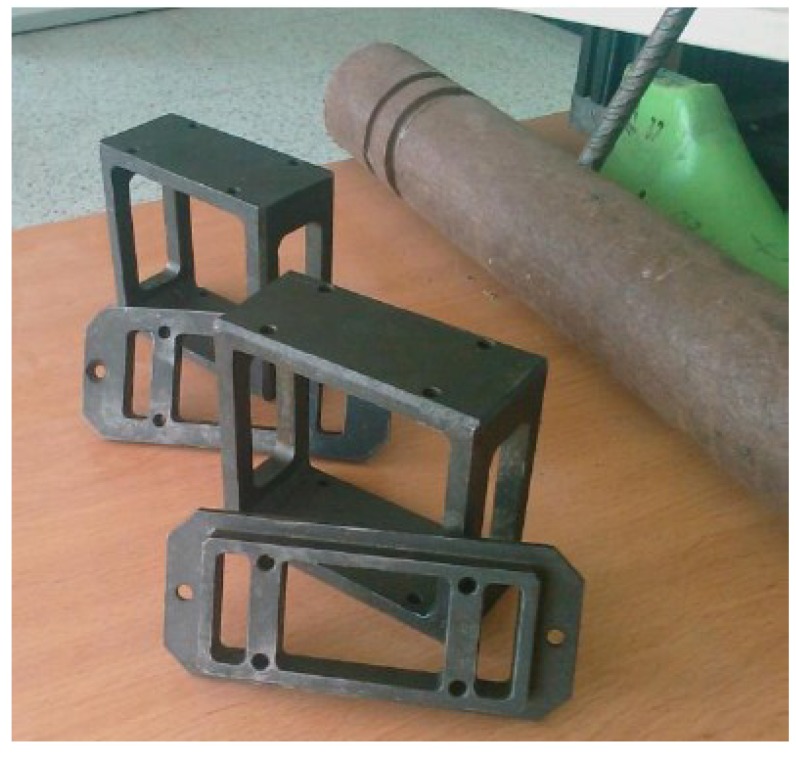
Pictures of the mask and the sliding guide.

A scheme of the process for selectively reinforcing the RPL beams is reported in Figure 4. At the beginning of mold filling the rods are introduced inside the mold, through the holes of the mask and the holes of the guide, as reported in Figure 4a. During mold filling, while the rods are held in position by the blocking system on the front surface of the mask, the guide is pushed forward by the molten polymer front. As the guide slides towards the back surface of the mold, the rods remain embedded in the polymer melt, as shown in Figure 4b. At the end of each molding cycle, the reinforced beam can be extracted, Figure 4c and the two metallic parts can be reused in the following cycle after cutting, Figure 4d. If so desired by alternative product design considerations, the layup of the rods can be changed by substituting the mask and the guide at the end of each molding cycle. As an example, in Figure 5, one picture of two masks and two sliding guides is reported. The two couples of tools are different, allowing for introduction of the rods at different distances from the half height of the beams.

In the framework of the Prowaste project, Solteco SL (Alfaro, Spain) was in charge of production of reinforced RPL. The extruder used to produce the beams is a model Kuhne 140/100, 25 L/D, equipped with forced feeding. The beams produced are 2960 mm long and 40 × 120 mm in cross section.

**Figure 6:**
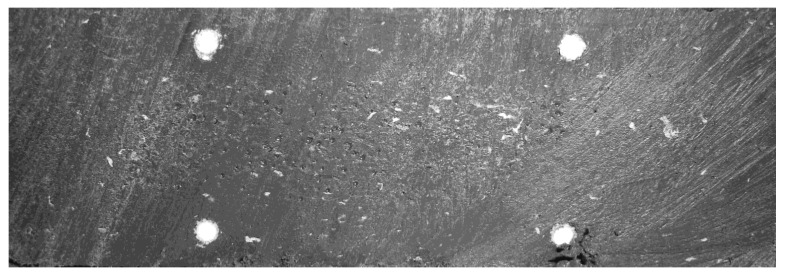
Picture of the cross section of the reinforced beam.

For each molding test, four pultruded rods were incorporated in the RPL beam, with a symmetrical layup with respect to the half-height of the profile. This was necessary to avoid any thermal distortion after extraction of the beam from the mold. The distance of the reinforcing rods from the external surface of the beam is 6 mm. Although this layup does not give the highest stiffening efficiency, the distance of 6 mm was chosen because it represents a minimum value necessary to obtain full rod wetting even in case of rod misalignment. A picture of the cross section of the reinforced beam obtained by the developed process is reported in Figure 6.

## 2. Materials and Methods

The polymer material used in the present work is a recycled plastic coming from solid urban waste. In the framework of the Prowaste project, Inserplasa SL (Industria Sevillana de Reciclados Plasticos) was in charge of the production of RPL.

**Figure 7:**
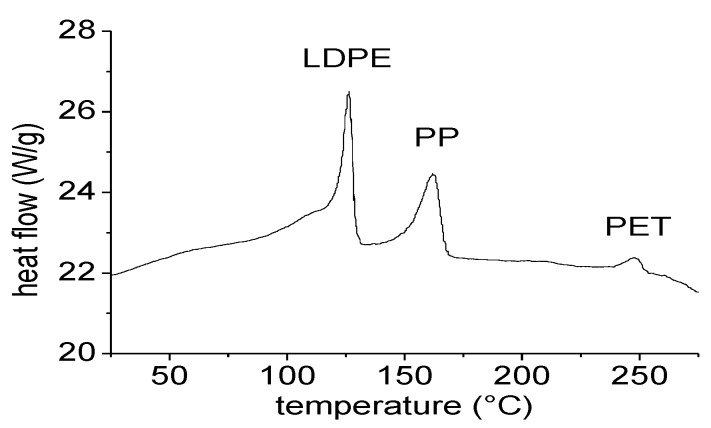
DSC analysis of RPL.

The RPL is obtained by manual sorting of plastics from solid urban waste. After removal of PET, HDPE and PP bottles, and of films of PP and PE bigger than about 20 × 30 cm, all the residues, mainly consisting of films of small dimensions, are collected as a mixed plastic. Such material mainly contains flexible and rigid PE and PP, but also small percentages of PET, escaped from the sorting stage. DSC analysis, reported in Figure 7, confirms that the material is mainly composed of LDPE, which melts in the range between 100 and 130 °C. Significant amounts of PP are also highlighted by the melting peak around 160 °C, as well as small traces of PET, which melts around 250 °C. Before extrusion, the material is simply milled to a size of about 8mm, and then pelletized at around 100 °C. No washing stage is foreseen.

**Figure 8:**
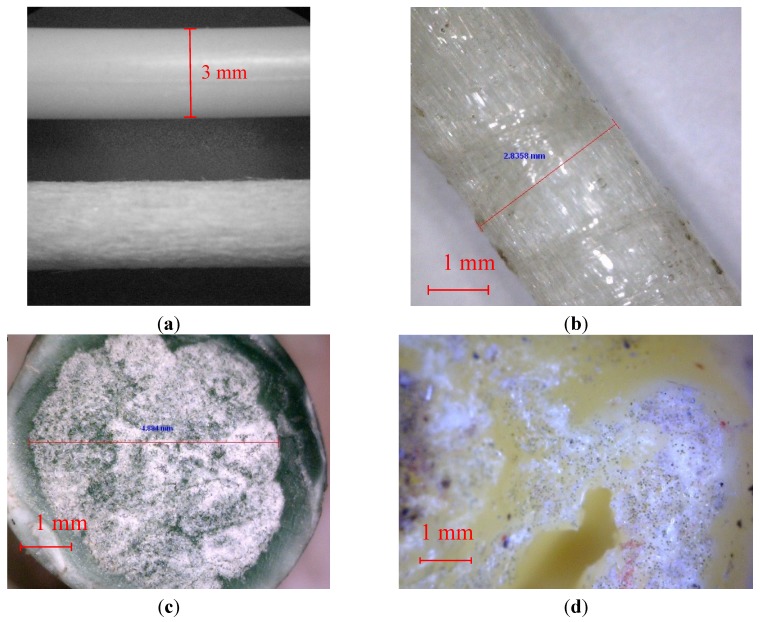
Micrographs of pultruded rods. (**a**) NEPTCO rods before (upper side) and after (bottom side) sandblasting; (**b**) POLYSTAL rods; (**c**) JONAM rods (cross-section surface); (**d**) JONAM rods after compression molding.

Different types of pultruded glass rods have been used:

- Pultruded rods for optical applications, characterized by a thermoset matrix and a very smooth surface, were purchased from NEPTCO. The mechanical and physical properties of the NEPTCO rods are reported in Table 2. Pultruded rods of 3 and 4 mm in diameter were used. In order to obtain a rough surface, the 3 mm rods were roughened by sandblasting. These rods are referred to as NEPTCO_s. The micrographs obtained by optical microscopy of the as received 3 mm rods and sandblasted 3 mm rods are reported in Figure 8a.
- Pultruded rods for civil engineering applications, characterized by a thermoset matrix and a rough surface, were kindly supplied by POLYSTAL Composites. The mechanical and physical properties of the POLYSTAL rods are reported in Table 2. A microscope image of POLYSTAL fibers is reported in Figure 8b. As it can be observed, the rods are characterized by the presence of fibers aligned in the longitudinal direction, as well as of fibers aligned tangentially. These fibers contribute to the increase of surface roughness, and therefore are expected to increase the adhesion strength.
- Pultruded rods based on a thermoplastic matrix (polypropylene, PP) were supplied by JONAM composites. The mechanical and physical properties of the POLYSTAL rods are reported in Table 2. The micrograph of the pultruded JONAM rods are reported in Figure 8c, showing that the rods are actually made of a glass fibers core, surrounded by a PP matrix. On the other hand, a higher magnification image, reported in Figure 8d, clearly shows that each bundle is actually surrounded by the matrix. Nevertheless, each bundle is completely dry, and no matrix is present inside. Therefore it is possible to conclude that macro-impregnation occurs, but no micro-impregnation [14].
- The pultruded carbon reinforced rods from BASF series Mbar Joint are carbon fiber/epoxy composites with a tensile modulus of 70 GPa.

**Table 2:** Physical and mechanical properties of pultruded rods.

Supplier	Reinforcing Fibers	Matrix	Diameter (mm)	Tensile Modulus (GPa)
NEPTCO	glass (85% wt)	thermoset	3, 4	50
POLYSTAL	glass (85% wt)	thermoset	3	50
JONAM	glass (51% wt)	thermoplastic	6	7
BASF	carbon	thermoset	6	70

Samples for rod pullout tests were obtained by a double stage compression molding process. At first, 9 × 60 × 200 mm samples of RPL were obtained by compression molding under 200 bar and a plate temperature of 30 °C, after preheating the material at 170 °C. Then, the RPL plate was divided in two parts. The pultruded rods were enclosed between the two RPL plates and compression molded at 200 bar and a plate temperature of 30 °C, after preheating of the materials at 170 °C.

Pull-out tests were carried out according to ASTM D1871-98 standard, using a Lloyd LR5K dynamometer. Rectangular specimens 30 mm × 9 mm × 40 mm were cut from the compression molded samples. Each specimen has a single reinforcing rod, which protrudes 30 mm from the cross section area of the plastic mass. The crosshead speed for the pull-out tests was 50 mm/min. Pull-out tests were used to determine the adhesion strength (τ_s_) between RPL and reinforcing rod, as:

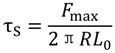
(4)
where *F*_max_ is the maximum load during the test, *R* is the rod diameter and *L*_0_ is the contact length between rod and polymer mass. For comparison purposes, pull-out tests were also performed on beams reinforced with pultruded carbon reinforced rods and steel rods.

Based on the results obtained from pull-out tests performed on the rods, thermosetting matrix based pultruded rods from NEPTCO were used for reinforcement of RPL. Four different prototypes were built. Beam NR was obtained by intrusion of the RPL without any reinforcement, beams R3 and R4 contained four rods of 3 mm and 4 mm in diameter, respectively, and beam R3s contained four 3 mm diameter sandblasted rods.

The specimens for flexural tests were obtained by cutting the beams along the length, to obtain samples with dimensions 40 mm × 120 mm × 800 mm. The span distance was 700 mm. For static flexural characterization, the sample was loaded in their middle section, with a crosshead speed of 10 mm/min, in accordance to ASTM D6109-97 standard. Cyclic flexural tests were performed between limits 0 to 1000 N, using a crosshead speed of 10 mm/min in both loading and unloading cycles. For flexural creep, test samples were placed in a oven and held at 50 °C for 1 h prior to testing. Then, the samples were loaded with a 240 N weight, and the deflection was recorded by means of a linear variable displacement transducer (LVDT) connected to a proprietary software, in accordance to ASTM D2990.

## 3. Results and Discussion

**Figure 9:**
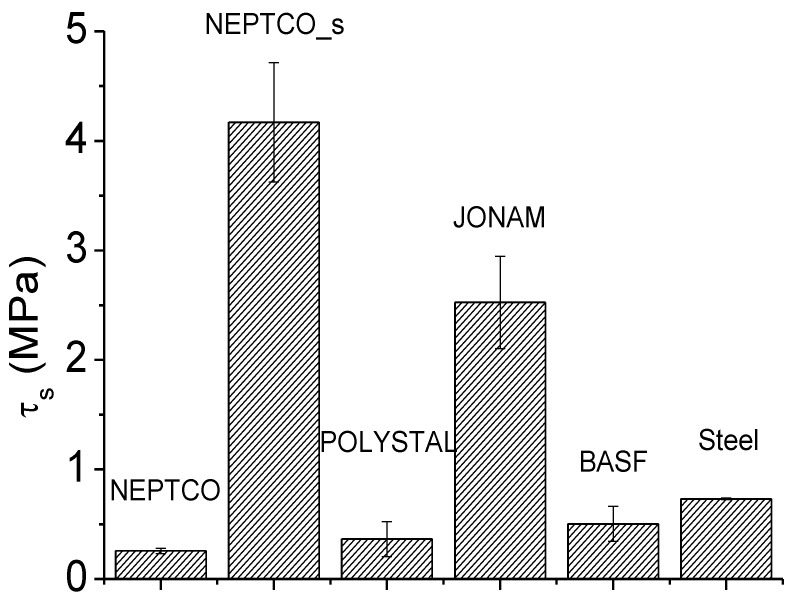
.Adhesion strength for different rods.

The results of the pull-out tests performed on the different rods are shown in Figure 9. The adhesion strength for the rods based on thermoset matrix, *i.e.*, NEPTCO and POLYSTAL, is quite poor, which is due to the fact that during processing, the matrix of the rod remains in the solid state, which prevents a good adhesion to the RPL. Most likely, the smooth surface of the rods (even those characterized by the presence of winded fibers, POLYSTAL) does not allow any mechanical gripping of the surface of the rod to RPL. On the other hand, the use of carbon and steel rods characterized by a rough surface, which are specifically designed for the building sector where adhesion to concrete is a key issue, allows for improvement of the adhesion to RPL. For the same reason, NEPCTO_s rods show an adhesion strength which is about 1 order of magnitude higher than that of the corresponding rods before sandblasting, NEPTCO. The difference is only a consequence of the different surface roughness of the materials. Instead, for JONAM rods, the improved adhesion can be attributed to the melting of the matrix during processing. The melting allows for some inter-diffusion at the interface between RPL and rod, which in turn involves an increase of the adhesion. In view of the results obtained, and also accounting for the cost considerations reported in Table 1, NEPTCO rods were used for the production of selectively reinforced RPL on the industrial plant.

**Figure 10:**
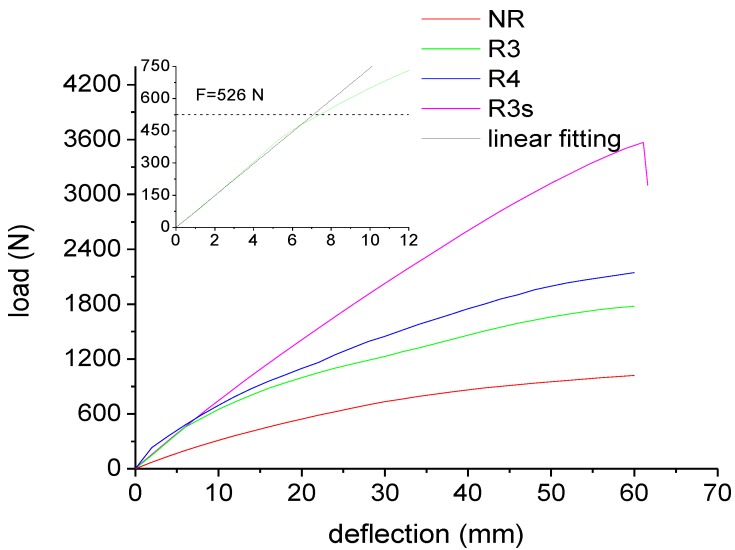
Load-deflection curves for plastic lumber beams.

The results from static flexural tests are reported in Figure 10. The behavior of the sample NR is highly non linear. However, from the slope of the curve at the origin, a modulus of 395 MPa can be calculated. When pultruded rods are incorporated in the plastic lumber beam, the flexural stiffness of the beam significantly increases.

By coupling Equations (1) and (2), the stiffness of the beams reinforced by the 3 mm and 4 mm diameter rods was estimated to be 74 and 100 N/mm, respectively. The linear prediction according to the estimated stiffness are reported in the inset of Figure 10 for beam R3, showing a very good agreement with the experimental curves. Figure 10 also shows a slope change for samples R3 and R4 at a load level of about 500 N. This discontinuity can be attributed to debonding of the rods as the shear stress exceeds the adhesion stress between matrix and rod. This occurs when:

τ_rz∣*R*_ = τ_s_ (5)

which, according to Equation (3), occurs when:

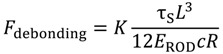
(6)
The value of *F* causing debonding, evaluated according to Equation (6), is 526 N, which is a value in a very close agreement to the experimental value, as evidenced in the inset of Figure 10.

For sample R3s, the properties at low stress levels are roughly the same of sample R3, indicating that the improvement of rod adhesion does not involve an improvement of the stiffness of the beam. In both cases, at low levels of deformation, the stress is transferred between the two phases by elastic shear, and there is perfect adhesion between the two phases. On the other hand, no discontinuity is observed in Figure 10 for the load-displacement curve of sample R3s, indicating the absence of debonding or slip effects at the interface between rod and RPL. Beam failure is observed under an applied load of about 3600 N. In correspondence of 3600 N load, Equation (3) allows to estimate a shear stress at the rod-RPL interface of 1.77 MPa, which is about 1/3 of the experimental value of adhesion strength reported in Figure 9 for sandblasted rods. This confirms that beam failure is not due to debonding at the rod/RPL interface. Indeed, the normal stress on the rod can be estimated to be [12]:

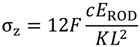
(7)
yielding a value of about 830 MPa, which is quite close to the tensile strength of the rods (about 1.4 GPa, as reported in the technical data sheet). This observation indicates that, for sample R3s, beam failure is due to rod tensile failure. For the samples R3 and R4, failure by flexural break did not occur even at displacements as high as 60 mm, as shown in Figure 10. This is the result of the very poor stress transfer between rod and polymer.

The results from flexural characterization suggest that failure of the reinforced RPL can occur due to two different phenomena:

(a) Debonding at the RPL-rod interface. This failure mode is very similar to yielding of ductile materials, since it involves a permanent plastic deformation, but does not involve a sudden decrease of the load bearing capacity. Debonding occurs when the force equals the value reported in Equation (7).
(b) Tensile failure of the rods. This failure mode is very similar to the rupture of a brittle material, since it involves a sudden decrease of the load bearing capacity of the beam. Tensile failure of the rods occurs when the normal stress on the rods equals their tensile strength, σ_R,ROD_, and the force attains a value given by inversion of Equation (7):

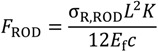
(8)

The lower value of the forces calculated according to Equations (6) and (8) determines the mode of failure of the reinforced beam. The results are reported in Table 3 for rods with 3 mm diameter in a beam 120 mm × 40 mm × 700 mm. As it can be observed, the NEPTCO rod-reinforced RPL fails due to debonding at the interface, and the same is likely to occur for POLYSTAL and JONAM rods. Despite this, the JONAM rods-reinforced RPL is likely to fail at much higher values of the applied load, as reported in Table 3, due to the much higher adhesion strength. In contrast, the JONAM rods-reinforced RPL is characterized by lower values of the stiffness, due to the lower modulus of JONAM rods compared to NEPTCO ones.

**Table 3:** Mode of failure of pultruded rod reinforced RPL.

Flexural properties of reinforced beams	NEPTCOϕ3	NEPTCO Sandblastedϕ3	POLYSTALϕ3	JONAMϕ3
*E*_R_ (GPa)	50	50	50	15
τ_S_ (MPa)	0.26	4.17	0.36	2.5
*F*_debonding_ (N)	526	8433	728	10,780
*F*_rod_ (N)	6067	6067	6067	12,834
Mechanism of failure	Debonding @ 526 N	Fiber tension @ 8433 N	Debonding @ 728 N	Debonding @ 10,780 N
Flexural stiffness (N/mm)	74	74	74	47

The results reported in Table 3 suggest that JONAM rods should be used when high load bearing capability is the most important design parameter, whereas NEPTCO rods should be chosen when a high stiffness is more relevant. The use of sandblasted NEPTCO rods allows for the obtainment of a very good compromise between high stiffness and high load bearing capacity. In this view, the choice between sandblasted and not sandblasted rods should only be made based on the economics of sandblasting, which is behind the scope of this work.

The results reported in Table 3, calculated according to Equation (7) and (8), do not account for the length of the beam. In fact, the results are relative to 700 mm long beams, which were used for mechanical characterization, though in most cases RPL beams can be as long as 2000 mm. In such cases, combining Equation (7) and (8) yields:

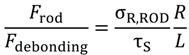
(9)
which gives a very useful tool for failure prediction. If the ratio 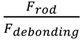 is higher than 1, failure occurs due to debonding, whereas rod tensile failure can occur when the ratio is lower than 1. For example, for JONAM rods, at a beam length of 700 mm, 
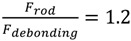
 indicates that failure occurs due to debonding, as reported in Table 3. On the other hand, for a beam length of 2000 m, 
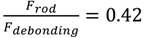
 indicates that failure is likely to occur due to rod tension.

**Figure 11:**
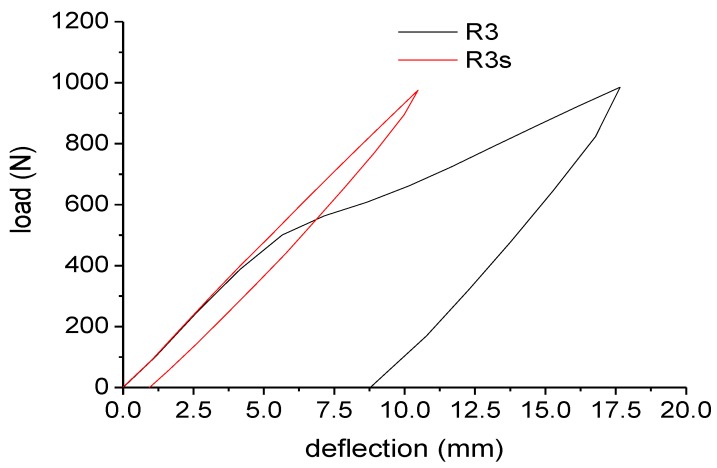
Cyclic loading tests for plastic lumber beams.

For a better understanding of the concept of debonding, and to highlight its analogy to the yielding of a ductile material, cycling loading tests were performed and the results are reported in Figure 11. The RPL reinforced with NEPTCO rods shows some important features. As also observed for the loading tests, a change of the slope in the curve at 530 N indicates the presence of debonding phenomena. After loading up to 1000 N, a significant permanent displacement (about 9.20 mm) is very similar to a plastic deformation for a ductile material. On the other hand, when RPL is reinforced with sandblasted NEPTCO rods, no change of the slope is observed during the loading stage. Consequently, the residual displacement after the unloading step is reduced to 0.9 mm. This is equivalent to a perfectly elastic behavior of the beam R3s between 0 and 1000 N.

Finally, it is possible to compare the present technology with other approaches which can be used for RPL stiffening:

(a) Addition of glass spheres, holding the same geometry of the beam.
(b) Increase of the thickness of the beam, holding the same material (unreinforced RPL).

For each layout of the reinforcing rods, it is possible to calculate the stiffness ratio, as the ratio between the stiffness of reinforced RPL, *K*_R_, and the stiffness of unreinforced RPL, *K*_NR_:

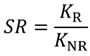
(10)


Addition of the glass spheres yields to an isotropic and homogeneous material, in which the reinforcement is also disposed in zones subjected to very low stresses. For each value of the stiffness ratio, the amount of glass spheres to be added can be obtained by inversion of the Halpin-Tsai equation [15]:

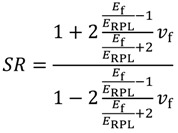
(11)
in which the elastic modulus of glass, *E*_F_, is 72 GPa, that of RPL, *E*_RPL_, is 395 MPa, and the aspect ratio of the reinforcing particles is assumed to be the unity. Assuming a plastic lumber density of 920 kg/m^3^ and a glass density of 2540 kg/m^3^, it is finally possible to calculate the corresponding weight of the beam.

**Figure 12:**
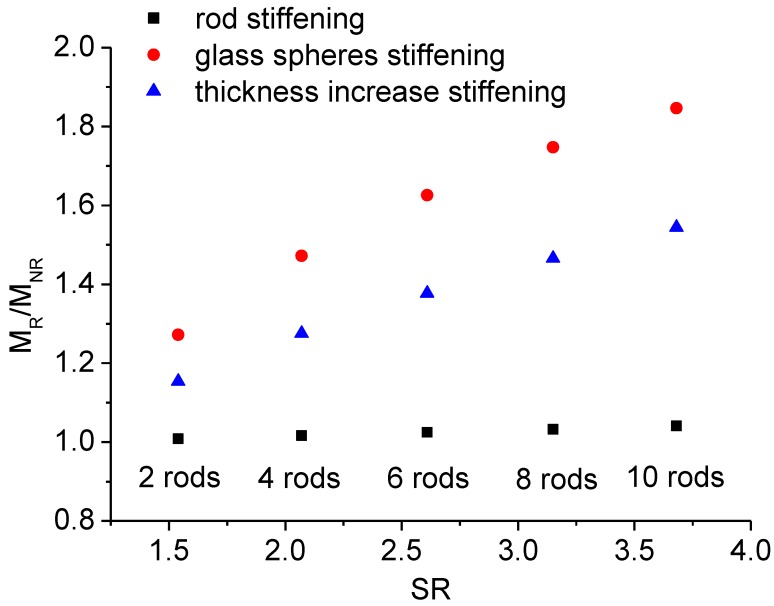
Comparison of the different stiffening approaches.

The equivalent increase of the thickness of the beam can be calculated starting by the modulus of the material:

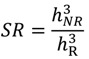
(12)
where: *h*_R_ is thickness of the pultruded rod reinforced beam, and *h*_NR_ the thickness of the unreinforced beam characterized by the same stiffness. The equivalent weight increase can be estimated considering the new geometry of the beam. The results are reported in Figure 12 for the three different methods. As it can be observed, the Prowaste concept allows to optimize the stiffening efficiency, reducing the weight increase to less than 4% for a layup in which 10 rods are disposed symmetrically, with a stiffening ratio *SR* = 3.7. Alternative methods, which introduce an uniform and homogeneous reinforcement, are less effective in increasing the stiffness, and involve a significant increase of the weight of the component.

**Figure 13:**
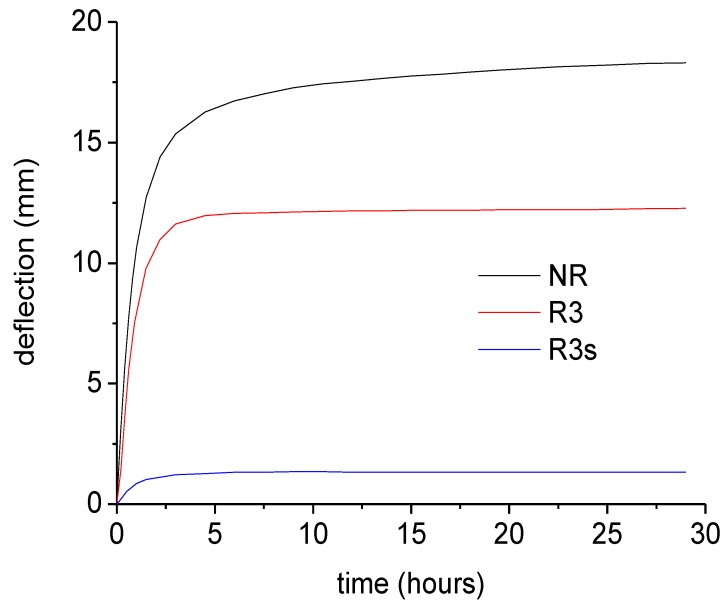
Creep curves for plastic lumber beams.

Finally, the creep curves for reinforced beams are reported in Figure 13. Compared to NR beam, the addition of the NEPTCO rods involves a reduction of the deflection of the beam by a factor of about 1.5. On the other hand, sandblasting of the rods has a dramatic effect, since it involves a reduction of the deflection measured after 25 h from 18.2 mm to 1.32 mm. This indicates that, in the case of R3 beam, slip at the rod/RPL interface plays a major role.

**Figure 14:**
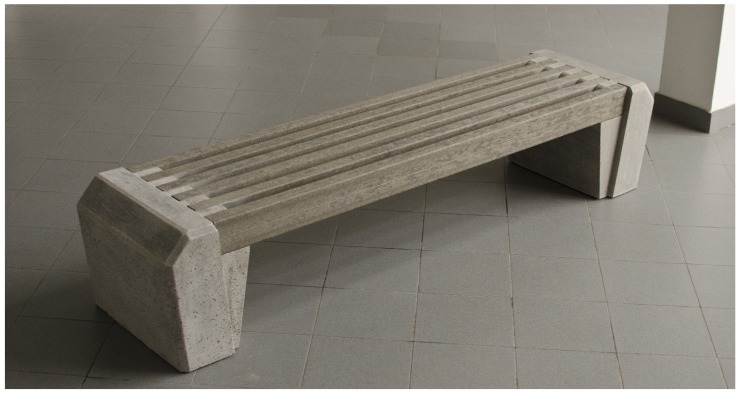
Picture of the bench produced with Prowaste profiles.

Finally, a picture of a bench produced by reinforced RPL is reported in Figure 14. In the framework of the Prowaste project, CETMA consortium (Brindisi, Italy) was in charge of the design and assembling of the bench. The introduction of the reinforcing rods allowed for the production of a bench characterized by a very long span (in this case almost 2000 mm) whereas for standard RPL span no longer than 800–900 mm are suggested. The bench was designed with 6 profiles fixed on 2 basements with a comb interlocking system, allowing easy assembly and different ways to seat.

## 4. Conclusions

The Prowaste project introduced a new design and process adaptation for the production of selectively reinforced recycled plastic lumber beams. The basic concept is based on the use of high aspect ratio reinforcing elements, placed in specific points of the cross section of the beams. Based on a primary evaluation of different classes of materials, pultruded glass rods are the best candidates for the proposed application. The process adaptation is based on the use of two metallic frames, the first one fixed on the front surface of the mold, and the second one capable to slide inside the mold with melt front during mold filling. The use of the two tools allows for disposal of the reinforcing rods parallel to the surface of RPL beam, at a specific distance from the half height of the beam cross section. Pull-out tests showed that thermosetting matrix pultruded rods are characterized by a very low adhesion strength to RPL. On the other hand, sandblasting can improve by one order of magnitude the adhesion strength. Instead, thermoplastic matrix rods, though being characterized by a low stiffness compared to thermosetting matrix rods, show a very good adhesion to RPL.

Flexural tests showed that the incorporation of pultruded glass rods in the RPL beams significantly improves the flexural stiffness. For thermosetting matrix rods having smooth surface, the poor interfacial adhesion is responsible of debonding at the polymer/rod interface when the interfacial stress overcomes adhesion strength. Debonding can be prevented by roughening the surface of the pultruded rod. This suggests that, based on the debonding behavior of reinforced RPL, the beam can behave either as a ductile or as a brittle material. In fact, debonding causes a significant non linearity, with the presence of significant plastic deformations, which is mechanically similar to yielding of a ductile material. In such circumstances, failure of the beam is not catastrophic, which is also very similar to ductile materials. On the other hand, a very high adhesion strength causes failure to occur due to tensile rupture of the rod, which in turn involves a sudden decrease of the load bearing capability. Similarly, a very high adhesion prevents debonding, and therefore plastic deformations. In such cases, the beam behaves like a brittle material.

A comparison with other stiffening approaches show that the Prowaste concept allows to minimize the weight increase, which is a key issue for RPL.

Finally, the Prowaste concept allowed for the production of a bench with an innovative design, in which, due to the high stiffness of the beam, the span was increased up to 2000 mm, which is a value much higher than that commonly used for RPL beams.
